# Supplementary material for: Increased and sex-selective avian predation of desert locusts Schistocerca gregaria treated with Metarhizium acridum
Source: PLoS One. 2021 Jan 4;16(1):e0244733. doi: 10.1371/journal.pone.0244733 (PMC7781369; doi:10.1371/journal.pone.0244733)
Supplement: S1 Appendix — (DOCX) [file pone.0244733.s001.docx]

**Appendix S1**. Birds recorded at Aghéliough (plus records from Arlit only).

1. Grey Heron *Ardea cinerea* (dead specimen only)
2. Cattle Egret *Bubulcus ibis* (Arlit only)
3. Egyptian Vulture *Neophron percnopterus* (Arlit only)
4. Black Kite *Milvus migrans* (September only)
5. Eurasian Marsh Harrier *Circus aeruginosus*
6. Booted Eagle *Hieraaetus pennatus* (September only)
7. Common Kestrel *Falco tinnunculus*
8. Lanner *Falco biarmicus abyssinicus and F. b. erlangeri*
9. Common Quail *Coturnix coturnix* (dead specimen only)
10. Nubian Bustard *Neotis nuba*
11. Cream-coloured Courser *Cursorius cursor*
12. Spotted Sandgrouse *Pterocles senegallus*
13. Feral Pigeon *Columba livia* (Arlit only)
14. African Collared Dove *Streptopelia roseogrisea*
15. Laughing Dove *Streptopelia senegalensis* (Arlit only)
16. Barn Owl *Tyto alba* (Arlit only)
17. Pallid Swift *Apus pallidus*
18. Common Swift *Apus apus*
19. Little Swift *Apus affinis*
20. Mottled Swift *Tachymarptis aequatorialis*
21. Hoopoe *Upupa epops senegalensis*
22. Singing Bush Lark *Mirafra cantillans*
23. Crested Lark *Galerida cristata*
24. Desert Lark *Ammomanes deserti*
25. Greater Short-toed Lark *Calendrella brachydactyla*
26. Black-crowned Sparrow-lark *Eremopterix nigriceps*
27. Greater Hoopoe Lark *Alaemon alaudipes*
28. Barn Swallow *Hirundo rustica*
29. Common House Martin *Delichon urbica*
30. Yellow Wagtail *Motacilla flava ? iberiae*
31. Rufous Scrub Robin *Cercotrichas galactotes*
32. Black-eared Wheatear *Oenanthe hispanica*
33. Desert Wheatear *Oenanthe deserti*
34. White-crowned Black Wheatear *Oenanthe leucopygia*
35. Eastern Olivaceous Warbler *Hippolais* *pallida*
36. Subalpine Warbler *Sylvia cantillans*
37. Cricket Warbler *Spiloptila clamans*
38. Brown babbler *Turdoides plebeja*
39. Fulvous Babbler *Turdoides fulva*
40. Southern Grey Shrike *Lanius meridionalis leucopygos*
41. Brown-necked Raven *Corvus ruficollis*
42. Chestnut-bellied Starling *Lamprotornis pulcher*
43. Sudan Golden Sparrow *Passer luteus*
44. Red-billed Firefinch *Lagonosticta senegalla* (Arlit only)
45. African Silverbill *Euodice cantans* (Arlit only)
46. White-rumped Seedeater *Serinus leucopygius*
